# Expression analysis of E-cadherin, Slug and GSK3β in invasive ductal carcinoma of breast

**DOI:** 10.1186/1471-2407-9-325

**Published:** 2009-09-14

**Authors:** Chandra P Prasad, Gayatri Rath, Sandeep Mathur, Dinesh Bhatnagar, Rajinder Parshad, Ranju Ralhan

**Affiliations:** 1 Department of Anatomy, Vardhman Mahavir Medical College and Safdarjung Hospital, New Delhi, India; 2 Department of Biochemistry, All India Institute of Medical Sciences, New Delhi, India; 3 Department of Pathology, All India Institute of Medical Sciences, New Delhi, India; 4 Department of Surgery, Vardhman Mahavir Medical College and Safdarjung Hospital, New Dehi, India; 5 Department of Surgery, All India Institute of Medical Sciences; New Delhi -110029, India; 6 Sonshine Family Centre for Head & Neck Disease, Mount Sinai Hospital, 600 University Avenue, Room 6-500, Toronto, Ontario M5G 1X5, Canada; 7 Department of Otolaryngology-Head and Neck Surgery, Mount Sinai Hospital, 600 University Avenue, Room 6-500, Toronto, Ontario M5G 1X5, Canada; 8 Department of Pathology and Laboratory Medicine, Mount Sinai Hospital, 600 University Avenue, Room 6-500, Toronto, Ontario M5G 1X5, Canada; 9 Department of Otolaryngology-Head and Neck Surgery, University of Toronto, Toronto, M5G 2N2, Canada

## Abstract

**Background:**

Cancer progression is linked to a partially dedifferentiated epithelial cell phenotype. The signaling pathways Wnt, Hedgehog, TGF-β and Notch have been implicated in experimental and developmental epithelial mesenchymal transition (EMT). Recent findings from our laboratory confirm that active Wnt/β-catenin signaling is critically involved in invasive ductal carcinomas (IDCs) of breast.

**Methods:**

In the current study, we analyzed the expression patterns and relationships between the key Wnt/β-catenin signaling components- E-cadherin, Slug and GSK3β in IDCs of breast.

**Results:**

Of the 98 IDCs analyzed, 53 (54%) showed loss/or reduced membranous staining of E-cadherin in tumor cells. Nuclear accumulation of Slug was observed in 33 (34%) IDCs examined. Loss or reduced level of cytoplasmic GSK3β expression was observed in 52/98 (53%) cases; while 34/98 (35%) tumors showed nuclear accumulation of GSK3β. Statistical analysis revealed associations of nuclear Slug expression with loss of membranous E-cadherin (p = 0.001); nuclear β-catenin (p = 0.001), and cytoplasmic β-catenin (p = 0.005), suggesting Slug mediated E-cadherin suppression via the activation of Wnt/β-catenin signaling pathway in IDCs. Our study also demonstrated significant correlation between GSK3β nuclear localization and tumor grade (p = 0.02), suggesting its association with tumor progression.

**Conclusion:**

The present study for the first time provided the clinical evidence in support of Wnt/β-catenin signaling upregulation in IDCs and key components of this pathway - E-cadherin, Slug and GSK3β with β-catenin in implementing EMT in these cells.

## Background

Breast cancer is a major cause of female mortality in the Western world. In India, it is the second most common cancer among females, while in the metropolitan cities; it ranks as the most common cancer [1]. The incidence rate is low as compared to the west with an age-adjusted incidence of 19.1 per 100,000 women and a crude incidence of 16.5 per 100,000 women [2]. The key biological processes such as embryonic development, tissue remodeling, restitution and wound repair, require epithelial cells to escape from the rigid structural constraints of the tissue architecture and adopt a phenotype more amenable to cell migration and movement. The highly conserved and fundamental process that achieves this morphogenetic transformation is known as epithelial-mesenchymal transition (EMT) [3,4]. The progression of tumors to invasive cancers and metastatic disease also involves localized occurrence of EMT [5,6].

Loss of E-cadherin mediated cell adhesion is one of the key mechanisms involved in metastatic conversion of epithelial cells and EMT [7,8]. Numerous studies have described a partial or complete loss of E-cadherin during cancer progression [9-12], which is often correlated with an unfavorable prognosis [13,14], confirming E-cadherin to be a caretaker of the epithelial state. One of the probable mechanisms involved in E-cadherin dysfunction, especially loss of its expression and consequent promotion of tumor progression is through β-catenin signaling. Several other mechanisms of E-cadherin downregulation have been described. Mutations have been found in the *CDH1 *gene in about 50% of lobular carcinomas of the breast [15], while ductal breast cancers show heterogeneous loss of E-cadherin expression, associated with epigenetic transcriptional downregulation. Analysis of *CDH1 *methylation in breast cancers and other tumor types has shown that aberrant hypermethylation of CpG islands in *CDH1 *promoter region often occurs prior to invasion, indicating it to be an early event in tumorigenesis [16]. Besides regulation of *CDH1 *by promoter methylation and/or genetic alterations, direct transcriptional control of *CDH1 *has emerged as an important regulatory mechanism of E-cadherin expression. The proteins Snail, Slug, and Twist have been recently characterized as transcriptional repressors of E-cadherin in breast carcinoma and are regulated by Wnt/β-catenin signaling [17,18]. Slug expression has been shown to correlate more strongly than snail expression with loss of E-cadherin in breast cancer cell lines, suggesting Slug to be a likely *in vivo *repressor of E-cadherin expression in breast carcinoma [19].

**Figure 1:**
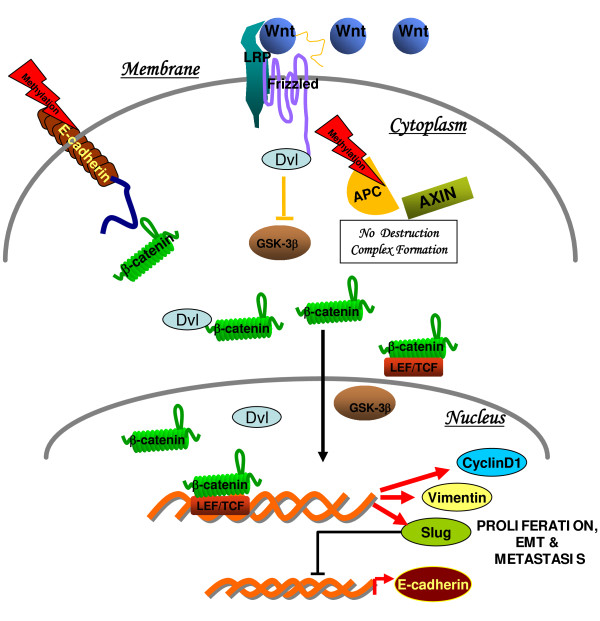
**Schematic diagram showing Wnt/β-catenin signaling in Invasive ductal carcinomas of breast**: On activation of Wnt signaling, disheveled (Dvl) prevents degradation of β-catenin, possibly through the recruitment of GBP/Frat-1, which in turn displaces GSK3β from the destruction complex. Adenomatous Polyposis Coli (APC), an important component of Wnt signaling was also found to downregulated by promoter methylation, as one of the mechanism [21]. Stabilized β-catenin enters the nucleus and associates with T cell factor (TCF)/lymphoid enhancer factor (LEF) transcription factors, which leads to the transcription of Wnt target genes such as *cyclin D1, vimentin and slug *[20,21]. Mechanisms attributed in the downregulation of E-cadherin are DNA methylation/or by transcriptional suppression via snail/or slug, thereby releasing membrane bound β-catenin into the cytosol [21].

The key components of EMT and Wnt pathways are schematically depicted in Figure 1. Recent results obtained in our laboratory showed that canonical Wnt signaling is one of the signaling pathways possibly involved in the control of the migration/invasive behavior of Invasive ductal carcinoma of breast (IDCs) [20,21]. We demonstrated the disorganization of E-cadherin-β-catenin complexes and the regulation of vimentin expression by β-catenin mediated pathway in IDCs, thereby supporting the notion that Wnt/β-catenin signaling is implicated in regulation of EMT [21].

E-cadherin, Slug and Glycogen synthase kinase 3β (GSK3β) play important roles in EMT transition via Wnt/β-catenin signaling. GSK3β resides at the junction of PI3K/AKT and Wnt/β-catenin/TCF survival pathways, thereby serving critical roles in cellular metabolism, growth and proliferation [22,23]. Under un-stimulated conditions, GSK3β pools are constitutively active, but they are phospho-inhibited upon PI3K/AKT or Wnt activation [24], resulting in cytoplasmic and nuclear accumulation of β-catenin.

Recent studies from our laboratory as well as by others substantiate the role of active Wnt/β-catenin pathway in proliferation and induction of EMT in tumor cells [5,20,21]. Taking our previous findings into consideration, the primary aim of this study was to determine the expression patterns of integral components of the canonical Wnt/β-catenin signaling- GSK3β, E-cadherin and Slug in IDCs and their associations with clinicopathological parameters for better understanding the biological and clinical relevance of Wnt/β-catenin pathway activation in sporadic breast cancer.

## Methods

### Tissue Specimens

Surgically resected specimens from untreated primary breast carcinomas and paired normal breast tissues were collected from 98 breast cancer patients enrolled in the Out Patients Department of Surgical Disciplines, Safdarjung Hospital and All India Institute of Medical Sciences (New Delhi, India), after approval of the study by Institutional Human Ethics Committee. Written consent was taken from all the patients enrolled in the study. The age of the patients ranged from 30-81 years with median age of 56 years. All the patients included in this study were invasive ductal breast carcinoma patients and their clinicopathological parameters are summarized in Table 1. In addition, 5 cases of Invasive lobular carcinomas (ILCs) were included to compare the expression of these proteins in ILCs and IDCs.

**Table 1:** Relationships between expression of E-cadherin, Slug and GSK3β in IDCs of breast: Correlation with clinicopathological parameters

Parameter	Total Cases (N)	E-Cad (Mem)			E-Cad (Cyto)			Slug (Nucl)			Gsk3β (Nucl)			Gsk3β (Cyto)		
		
		-	+	*P*	-	+	*P*	-	+	*P*	-	+	*P*	-	+	*P*
	
	98	53	45		46	52		65	33		64	34		52	46	
Age																
ὄ 50	62	30	32	.148	24	38	0.06	40	22	.663	44	18	0.131	30	32	.294
>50	36	23	13		22	14		25	11		20	16		22	14	

Menopausal status																
Pre-	35	19	16	1.00	12	23	0.091	22	13	.658	27	8	.079	19	16	1.00
Post-	63	34	29		34	29		43	20		37	26		33	30	

Tumor grade																
T_1_+T_2_	53	24	29	.060	21	32	0.155	29	24	.01*^1^	29	24	.02*^2^	26	27	.422
T_3_+T_4_	45	29	16		25	20		36	9		35	10		26	19	

Lymphatic involvement																
N_0_	36	19	17	1.00	18	18	0.679	22	14	.507	20	16	0.131	18	18	.675
N_1-2_	62	34	28		28	34		43	19		44	18		34	28	

Grade																
I+II	56	27	29	.221	22	34	0.103	33	23	.087	32	24	.05*^3^	31	25	.684
III+IV	42	26	16		24	18		32	10		32	10		21	21	

ER																
+ve	28	17	11	.502	13	15	1.00	15	13	.103	13	15	.019*^4^	17	11	.377
-ve	70	36	34		33	37		50	20		51	19		35	35	

*TNM Classification on basis of AJCC classification [46]. **Abbreviations**: E-cad, E-cadherin; Cyto, Cytoplasmic; Nucl, Nuclear and Mem, Membrane. *^1^p = 0.01; OR = 0.191 [95% CI = 0.122-0.75]; *^2^p = 0.02; OR = 0.3 [95%CI = 0.142-0.838]; *^3^p = 0.05; OR = 0.417 [95%CI = 0.172-1.01]; *^4^p = 0.019; OR = 3.09 [95%CI= 1.24-7.09]. * p values were calculated using Fischer exact test. p < 0.05 was considered as significant and are given in the table. Analysis of relationships between E-cadherin, Slug and GSK3β expression in IDCs showed the following significant associations: **E-cad (Mem)^-^/Slug (Nucl)^+^: p = 0.001; OR = 4.49 [95% C.I = 1.82-11.8]; E-cad (Cyto)^+^/E-cad (Mem)^-^: p = 0.016; OR = 2.82 [95% C.I = 1.23-6.44]; E-cad (Cyto)^+^/Slug (Nucl)^+^: p = 0.031; OR = 2.88 [95% C.I = 1.17-6.95]; GSK3β (Cyto)^+^/GSK3β (Nucl)^+^: p = 0.012; OR = 3.06 [95% CI = 1.28-7.27]**.

### Immunohistochemical analysis of E-cadherin, Slug and GSK3β

Immunohistochemical analysis of E-cadherin, Slug and GSK3β was carried out using paraffin embedded tissue sections as described by us previously [20,21]. E-cadherin monoclonal antibody (sc-8426) and Slug polyclonal antibody (sc-15391) were purchased from Santa Cruz Biotechnology Inc. (Santa Cruz, CA). Monoclonal GSK3β antibody (Cat. No.610201) was procured from BD Biosciences (San Jose, CA). The monoclonal antibodies were used at 1:100 dilution and the polyclonal antibody was used at 1:50 dilution. In the negative controls, the primary antibody was replaced by isotype- specific IgG.

### Scoring Criteria for Immunohistochemical Staining

Evaluation of immunohistochemistry was carried out by two investigators independently (CPP, SM). Tumors were classified based on the percentage of cells showing immunoreactivity. The immunostained slides were scored as per the following criteria for all the three proteins: no detectable staining, negative; +, ≤ 10%; ++, 10-50%; +++, >50% of positive tumor cells. Tumors were regarded as immunopositive if >10% of tumor cells showed immunoreactivity. For the transcription factor Slug, detectable immunoreactivity in nuclear region was defined as positive expression [18]. Expression of E-cadherin protein was localized in membrane/cytoplasm/or both in IDCs, both membranous and cytoplasmic expression were considered as event [25]. Nuclear/cytoplasmic staining was considered positive for GSK3β [26].

### Immunoblot analysis of E-cadherin, GSK3β and Slug in breast normal tissues and IDCs

Tissue lysates were prepared from 4 normal breast tissues (collected from 5-7 cm away of tumor periphery from radical mastectomy specimen) and 4 IDCs. Frozen tissue samples were homogenized and lysed in RIPA buffer containing 1× protease inhibitor cocktail. Protein concentrations were determined using the Bradford assay (Sigma Chemicals, Bangalore, India) and equal amounts of proteins (80 μg/lane) from tissue lysates were electrophoresed in 10% sodium dodecyl sulfate-polyacrylamide gels and then transferred onto Polyvinylidenedifluoride (PVDF) membranes. After blotting in 5% non-fat dry milk in Tris-buffered saline, blots were incubated with anti-E-cadherin antibody (1:500 dilution), anti-GSK3β antibody (1:1000 dilution), and anti-Slug antibody (1:200 dilution) at 4°C overnight. Membranes were incubated with secondary antibody, HRP-conjugated rabbit/mouse anti-IgG (DAKO Cytomation, Denmark), diluted at an appropriate dilution in 1% BSA, for 2 hrs at room temperature. Protein bands were visualized on X-ray film using an enhanced chemiluminescence system (ECL, Santa Cruz Biotechnology, CA).

### Statistical Analysis

All the statistical analysis were performed using SPSS 10.0 for Windows. (SPSS Inc., Chicago, IL). Chi-Square test and Fischer's exact test (2-sided) were performed for determining the correlation between clinicopathological parameters and protein expression. Results were considered significant when p value was < 0.05.

## Results

### Immunohistochemical analysis of E-cadherin, GSK3β and Slug in Invasive ductal carcinoma of breast

**Figure 2:**
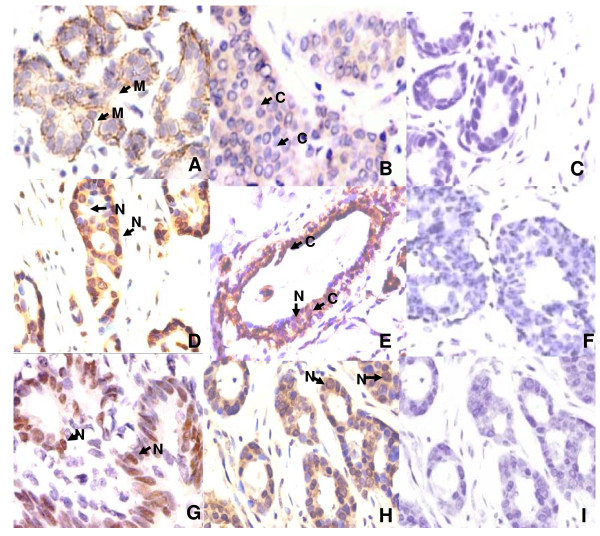
**Expression of E-cadherin, Slug and GSK3β proteins in invasive ductal carcinomas of breast**. **(A) **Strong membranous E-cadherin expression in normal breast tissue (**B**) Loss of E-cadherin membranous localization in tumor cells. **(C) **Loss of Slug protein in normal breast (**D**) Nuclear accumulation of Slug in IDC **(E) **GSK3β expression in normal breast epithelium (**F**) IDC showing loss cytoplasmic GSK3β **(G) **GSK3β nuclear localization in tumor cells; and **(H&I) **Nuclear accumulation of Slug and loss of E-cadherin immunostaining examined on the adjacent sections of the same tumor. (**A**-**I**, original magnification × 400). Arrows show Membranous (M), Nuclear (N) and cytoplasmic (C) localization of the proteins.

The results of immunohistochemical analysis of E-cadherin, GSK3β and Slug expression in IDCs are summarized in Table 1. Strong membranous E-cadherin immunostaining was observed in normal breast tissues (Figure 2A). Of the 98 IDCs examined, 53/98 (54%) showed loss/or reduced membranous staining of E-cadherin in tumor cells; 52/98 (53%) of IDCs showed cytoplasmic accumulation of the protein (Figure 2B). No association was found between loss of membranous/or cytoplasmic accumulation of E-cadherin expression with any of the clinicopathological parameters (Table 1).

Slug was expressed in 33 of 98 (34%) IDCs examined (Table 1), while the paired histological normal breast tissues did not show detectable levels of Slug protein (Figure 2C). Expression of Slug protein was observed in cytoplasm/nucleus/or both, however, only nuclear expression was considered as immunopositive for Slug (Figure 2D). Significant inverse association was observed between nuclear accumulation of Slug and tumor stage [p = 0.01; OR = 0.191; (95% CI = 0.122-0.75)] in IDCs (Table 1).

GSK3β expression was localized in the cytoplasm/or nucleus of epithelial cells in normal breast tissues (Figure 2E). In comparison loss/or reduced level of cytoplasmic GSK3β expression was observed in 52/98 (53%) breast carcinomas, while 34/98 (35%) of IDCs showed nuclear accumulation of GSK3β (Figures 1F &1G respectively). Nuclear GSK3β was inversely associated with tumor stage [p = 0.02; OR = 0.3; (95% CI = 0.142-0.838)] and histological grading [p = 0.05; OR = 0.417 (95% C.I = 0.172-1.01)] in IDCs (Table 1). Positive association was also observed between GSK3β nuclear expression and ERα receptor positivity [p = 0.019; OR = 3.09; (95% CI = 1.24-7.09)]. Furthermore, immunohistochemical confirmed cases of IDCs and normal breast tissues were revalidated by Western blotting for protein expression. Western Blot analysis independently confirmed the immunohistochemical findings of E-cadherin, GSK3β and Slug proteins expressions in IDCs of breast and paired normal breast tissues (data not shown).

### Expression patterns of E-cadherin, GSK3β and Slug in Invasive lobular carcinomas (ILCs) of breast

Five sections of histological confirmed Invasive lobular carcinoma (ILC) of breast were also analyzed for E-cadherin, Slug and GSK3β staining. Four of 5 ILCs showed complete loss of E-cadherin protein, as shown in Figure 2A and 2B. Distinct slug nuclear localization was observed in 3 of the 5 ILCs analyzed (Figure 2C); whereas, all the 5 ILCs showed no detectable expression of GSK3β protein (Figure 2D).

### Relationship between E-cadherin, GSK3β and Slug expression in IDCs

Significant association was observed between nuclear expression of Slug and loss of membranous E-cadherin [p = 0.001; OR = 4.49 (95% C.I = 1.82-11.8)]. Figure 2H and 2I show nuclear accumulation of Slug and loss of E-cadherin immunostaining respectively examined on consecutive sections of the same tumor in representative cases, supporting the association between these proteins found by statistical analysis. Positive associations were also observed between cytoplasmic accumulation E-cadherin protein and loss of membranous E-cadherin [p = 0.016; OR = 2.82 (95% C.I = 1.23-6.44)] as well as with nuclear Slug [p = 0.031; OR = 2.88 (95% C.I = 1.17-6.95)]. Positive association was also observed between cytoplasmic and nuclear accumulation of GSK3β [p = 0.012; OR = 3.06 (95% C.I = 1.28-7.27)] (Table 1).

### Association of β-catenin with E-cadherin, Slug and GSK3β in IDCs

The expression and sub-cellular distribution of β-catenin in these IDCs has been reported by us earlier in the same cohort [20]. Here in statistical analysis showed significant associations between β-catenin expression and E-cadherin, Slug and GSK3β, as summarized in Table 2. Loss of membranous E-cadherin was significantly associated with loss of membranous β-catenin [p = 0.0001; OR = 5.59 (95% C.I = 2.21-14.10)]. Positive association was observed between cytoplasmic localization of E-cadherin and nuclear β-catenin [p = 0.046; OR = 2.33 (95% CI = 1.03-5.25)]. Nuclear accumulation of Slug showed significant association with nuclear β-catenin [p = 0.001; OR = 4.87 (95% C.I = 1.94-12.21)]; cytoplasmic β-catenin accumulation [p = 0.005; OR = 3.67 (95% C.I = 1.53-8.83)] and β-catenin membranous loss [p = 0.012; OR = 3.25 (95% C.I = 1.34-7.89)].

**Table 2:** Analysis of relationships between E-cadherin, Slug and GSK3β expression with β-catenin expression in IDCs.

Parameter	Total Cases (N)	E-Cad (Mem)			E-Cad (Cyto)			Slug (Nucl)			Gsk3β (Nucl)			Gsk3β (Cyto)		
		
		-	+	P	-	+	P	-	+	P	-	+	P	-	+	P
	
	98	53	45		46	52		65	33		64	34		52	46	
β-catenin (Mem.)																
Positive	33	9	24	.0001*^1^	12	21	.199	16	17	0.012*^3^	19	14	0.270	19	14	0.669
Negative	65	44	21		34	31		49	16		45	20		33	32	

β-catenin (Cyto.)																
Positive	42	20	22	0.309	18	24	0.543	21	21	0.005*^4^	24	18	0.198	17	25	0.070
Negative	56	33	23		28	28		44	12		40	16		35	21	

β-catenin (Nucl.)																
Positive	47	25	22	1.000	17	30	0.046*^2^	23	24	0.001*^5^	28	19	0.292	20	27	0.068
Negative	51	28	23		29	22		42	9		36	15		32	19	

**Abbreviations**: E-cad, E-cadherin; Cyto, Cytoplasmic; Nucl, Nuclear and Mem, Membrane.*^1^β-cat (Mem)^-^/E-cad (Mem)^-^: p = 0.0001; OR = 5.59 [95% C.I = 2.21-14.1]; *^2^β-cat (Nucl)^+^/E-cad (Cyto)^+^: p = 0.046; OR = 2.33 [95% CI = 1.03-5.25]; *^3^β-cat (Mem)^-^/Slug (Nucl)^+^: p = 0.012; OR = 3.25 [95% CI = 1.34-7.89]; *^4^β-cat (Cyto)^+^/Slug (Nucl)^+^: p = 0.005; OR = 3.67 [95% C.I = 1.53-8.83]; *^5^β-cat (Nucl)^+^/Slug (Nucl)^+^: p = 0.001; OR = 4.87 [95% C.I = 1.94-12.21].

Of the 98 IDCs analyzed, 33 tumors showed nuclear accumulation of Slug. Of these 33 tumors, 17 (51%) IDCs showed nuclear localization of β-catenin protein (p ὄ 0.01); while 23 (70%) cases showed loss of membranous E-cadherin expression (p ὄ 0.01). Twelve of 33 (36%) IDCs showed both nuclear localization of β-catenin protein and loss of membranous E-cadherin.

## Discussion

Recent studies demonstrate that canonical Wnt signaling pathway is associated with both stem cell and tumor cell development [27-29]. In many tissues, where stem cell attributes are under the control of Wnt pathway, aberrant activation of this pathway results in tumor formation [30]. Our current findings support the accumulating evidence that hyperactive Wnt signaling is associated with development and progression of human breast cancer [20,21,31]. Herein, we provide clinical evidence to demonstrate that alterations in expression of the key components of Wnt/β-catenin pathway- E-cadherin, GSK3β and Slug occur in the pathogenesis of IDCs.

During EMT, the epithelial cells acquire fibroblast-like properties and show reduced intercellular adhesion and increased motility; this process is associated with functional loss of E-cadherin [32,33]. Further, down regulation of E-cadherin is the key step towards invasive phase of cancer progression; promoter methylation/transcriptional repression are mechanisms largely responsible for loss of E-cadherin expression in IDCs [34-36]. Recent data from our laboratory suggests that absence of E-cadherin is partly attributed to promoter methylation of *CDH1 *in IDCs; *CDH1 *hypermethylation frequency was found to be 50% in IDCs [21]. Taking into account the two major histological subtypes of breast cancer, however, different modes of E-cadherin expression modulation have been found. While infiltrating ductal breast cancers mostly show no or only heterogeneously reduced E-cadherin expression, infiltrative lobular breast carcinomas (ILC) are, in most cases (95%), completely E-cadherin-negative [9-12].

In the present study, we observed loss/or reduced expression of E-cadherin in 54% (53/98) of IDCs. Similar E-cadherin loss was reported in 85% cases in a series of 71 ductal carcinomas and correlated with promoter methylation of *CDH1 *[25]. In our previous study in the same patient cohort, we analyzed the expression patterns of β-catenin, Disheveled and CyclinD1. Of the 98 IDCs analyzed, loss of cell surface β-catenin was observed in 66% cases, whereas nuclear expression was observed in 44% tumors [20]. In the current study, we found significant positive association between membranous E-cadherin and β-catenin loss (p = 0.0001) in IDCs. On this basis, we postulate that E-cadherin loss promotes tumorigenesis by effectively releasing membrane bound β-catenin into the cytosol, there by stimulating the canonical Wnt signaling.

**Figure 3:**
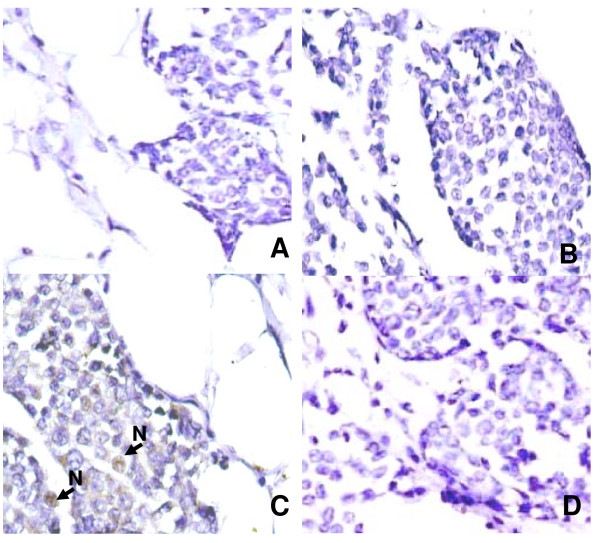
**Expression pattern of E-cadherin, Slug and GSK3β proteins in invasive lobular carcinomas of breast**. **(A& B) **Invasive lobular carcinomas showing loss of E-cadherin staining **(C) **Invasive lobular carcinoma showing nuclear staining for Slug **(D) **ILC showing complete loss of GSK3β protein. (**A**-**D**, original magnification × 400).

Slug mediated loss of E-cadherin expression in IDCs is another finding of our study. Although several transcription factors including Snail and Slug have been implicated in E-cadherin repression, herein we have analyzed the expression of Slug only, because it has been proposed to be a likely *in vivo *repressor of E-cadherin as compared to snail in breast carcinomas [19,37]. Nuclear accumulation of Slug was observed in 33 of 98 (34%) IDCs, though lower than reported in a previous study [17], and correlated inversely with tumor grade (p = 0.01) and loss of membranous E-cadherin expression (p = 0.033) in IDCs. The association between E-cadherin membrane loss and its cytoplasmic accumulation with nuclear Slug prompts us to speculate that Slug may act as a transcriptional suppressor of E-cadherin and regulate its cellular turnover in IDCs. Furthermore, significant associations between nuclear and cytoplasmic β-catenin and Slug (p = 0.001; p = 0.005), underscores the importance of β-catenin mediated regulation of Slug in invasive ductal carcinoma of breast. In addition, we also performed Immunohistochemistry for E-cadherin and Slug proteins on 5 histologically confirmed cases of Invasive lobular carcinomas (Figure 3). Our result is in the agreement with the previous studies, showing total loss of E-cadherin protein in ILCs [11,12]. However, we did find nuclear accumulation of slug in 3 of 5 ILCs. Till date there is no informative study on Slug protein in ILCs, therefore future studies are warranted in this specific area for defining/or elucidating the role of Slug in ILCs.

The other important component of Wnt pathway investigated in our study is GSK3β, a multikinase involved in Wnt, Akt and Hedgehog pathways, all of which are involved in determination of cell fate and morphology; inhibition of GSK3β activity or expression results in bonafide EMT [24,38,39]. We observed loss of GSK3β protein in 53% of IDCs, suggesting endogenous suppression of the GSK3β, either due to Wnt or PI3K-kinase, which are frequently activated in IDCs [20,40]. In the canonical Wnt/β-catenin pathway, GSK-3β activity in the destruction complex is inhibited through a yet unclear process, leading to the accumulation of β-catenin that translocates to the nucleus and activates transcription by TCF/LEF transcription factors. In our study, a subset of IDCs (35%) showed nuclear accumulation of GSK3β protein. Other plausible reason for nuclear GSK3β accumulation may be an additional regulation inside the cell and complete inhibition of GSK3β may require activation of multiple signaling pathways simultaneously. Further studies on the expression profiles of pGSK3β and pAkt, using phospho-specific antibodies will certainly help in elucidating the role of GSK3β regulation in Invasive ductal carcinomas of breast. However, these mechanisms need to be proven in future studies. We also observed an inverse association of nuclear GSK3β with tumor grade (p = 0.02), suggesting that the initial tumor development probably requires a rapid and effective repression of GSK3β and stabilization of Slug, thereby inhibiting the expression of E-cadherin. Interestingly, nuclear GSK3β showed positive association with ERα expression (p = 0.019), suggesting that GSK3β may regulate the estrogen receptor mediated transcription in subsets of IDCs.

In a recent study, we demonstrated the relationship of loss of E-cadherin and APC proteins with activation of Wnt/β-catenin signaling driving EMT [21]. We demonstrated therein, that apart from β-catenin, Disheveled also regulates the expression of Vimentin, establishing direct association of Wnt/β-catenin signaling with EMT. Continuing our focus on Wnt/β-catenin signaling and EMT, in the present study we found various relationships among EMT regulators like β-catenin, E-cadherin and Slug in IDCs (Table 2).

Our current findings also support the concept that generation of cancer stem cells (CSCs) - the acquisition of the stemness and tumorigenic characters is driven by induction of EMT [41]. In breast cancer, CD44^+^/CD24^- ^population harbors stem cell properties. These CD44^+^/CD24^- ^cells, express low or undetectable levels of epithelial markers (E-cadherin and β-catenin) and high levels of mesenchymal markers (vimentin and fibronectin), suggesting that these cells have undergone EMT [42]. Expression of EMT-inducing factors, such as E-cadherin, β-catenin and Slug, has been shown to be associated with breast tumor recurrence and metastasis [37,43,44]. Importantly, Wnt signaling has been recently established to serve as a molecular link between self renewal, EMT, and metastasis in basal-like breast cancers supporting our clinical findings [45]. In the presence of Wnt signals, β-catenin has been proposed to partner with TCF/LEF to activate target genes, such as Slug and Twist which promote an EMT, repress differentiation, increase tumor seeding and metastasis. Thus Wnt signaling effects Slug and Twist thereby regulating cell-cell adhesion and EMT; it can also connect EMT with cell fate and differentiation. Taken together, we speculate that E-cadherin, Slug and GSK3β could be exploited as markers and pharmacologic or antibody-based therapies targeting the Wnt pathway components, which may not only improve the management of breast cancer, but also affect tumor recurrence and/or metastasis.

## Conclusion

In the present study, we provide clinical evidence in support of up-regulation of Wnt/β-catenin signaling in invasive ductal carcinoma of breast and key components of this signaling pathway such as E-cadherin, Slug, GSK3β and β-catenin to be associated with Epithelial Mesenchymal Transition (EMT) process and pathogenesis of IDCs. Moreover, a better understanding of the pathways (such as the Wnt/β-catenin signaling pathway) that trigger EMT and cancer cell self renewal (cancer stem cells) might lead to new therapeutic approaches for cancer patients by developing molecular tools that interfere with them.

## Abbreviations

IDC: Invasive Ductal Carcinoma; EMT: Epithelial Mesenchymal Transition; PI3K: Phosphoinositide 3-kinase; GSK3β: Glycogen Synthase Kinase3β; Dvl: Disheveled.

## Competing interests

The authors declare that they have no competing interests.

## Authors' contributions

**CPP **planned, set up the experiments, collected the data, analyzed and interpreted the results and drafted the manuscript. **GR **participated in the study design, interpretation of the results and in editorial support. **SM **conducted histopathological evaluation of all clinical specimens and assessment of immunohistochemical staining data. **DB **enrolled the patients in the study, provided the clinical specimens, patient data, follow up and clinical knowledge. **RP **enrolled the patients in the study, provided the clinical specimens, patient data and clinical knowledge. **RR **planned, supervised, provided financial and technical support for the study and writing of the manuscript. All authors have read and approved the final manuscript.

## Conflict of interests

The authors declare that they have no competing interests.

## Pre-publication history

The pre-publication history for this paper can be accessed here:

http://www.biomedcentral.com/1471-2407/9/325/prepub
